# Effect of Short Fibers on Fracture Properties of Epoxy-Based Polymer Concrete Exposed to High Temperatures

**DOI:** 10.3390/polym15051078

**Published:** 2023-02-21

**Authors:** Oussama Elalaoui

**Affiliations:** 1 Department of Civil and Environmental Engineering, College of Engineering, Majmaah University, Al-Majmaah 11952, Saudi Arabia; o.elalaoui@mu.edu.sa or elalaoui_o@yahoo.fr; 2 Civil Engineering Laboratory, National Engineering School of Tunis, University of El Manar, BP 37, Tunis 1002, Tunisia

**Keywords:** polymer concrete, short fibers, fracture properties, thermal resistance, porosity

## Abstract

Recently, polymer concrete (PC) has been widely used in many civil engineering applications. PC shows superiority in major physical, mechanical, and fracture properties comparing to ordinary Portland cement concrete. Despite many suitable characteristics of thermosetting resins related to processing, the thermal resistance of polymer concrete composite is relatively low. This study aims to investigate the effect of incorporating short fibers on mechanical and fracture properties of PC under different ranges of high temperatures. Short carbon and polypropylene fibers were added randomly at a rate of 1 and 2% by the total weight of the PC composite. The exposure temperatures cycles were ranged between 23 to 250 °C. Various tests were conducted including flexure strength, elastic modulus, toughness, tensile crack opening, density, and porosity to evaluate the effect of addition of short fibers on fracture properties of PC. The results show that the inclusion of short fiber lead to an increase in the load carrying capacity of PC by an average of 24% and limits the crack propagation. On the other hand, the enhancement of fracture properties of based PC containing short fibers is vanished at high temperature (250 °C), but still more efficient than ordinary cement concrete. This work could lead to broader applications of polymer concrete exposed to high temperatures.

## 1. Introduction

Polymer concrete (PC), one of the three types of polymer concrete type, is formed by completely substituting Portland cement with polymer binders. The two other types are polymer-impregnated concrete (PIC), which consists of a hardened Portland cement concrete impregnated with a low-viscosity monomer polymerized in situ, and the second one is the polymer cement concrete (PCC) which is produced by incorporating a monomer or polymer in a Portland cement concrete mix and polymerized after placing concrete [1].

The polymer binder used in PC is acting through a polymerization process to bond the solid particles of concrete [2,3]. PC, whether reinforced or non-reinforced, shows superiority in major physical, mechanical, and fracture properties when compared to ordinary Portland cement. These properties include high mechanical strength, rapid hardening, and short curing time, which are very beneficial for precast element production or partitions with high toughness. [4]. In addition, PC is attributed with fast demolding, minimum cracking, higher mechanical strength, good bonding to old substrates, better abrasion resistance, chemical resistance, and less maintenance in comparison to Portland cement concrete [5,6]. This explains the quick development of using PC in many civil engineering applications [7].

Various types of resins have been used in the production of polymer concrete such as epoxy [8,9], unsaturated polyester [10,11], acrylics and vinyl-ester, poly acrylate, polypropylene, furan-based resin, and other polymers [12,13]. However, the formed composites incorporating these materials are brittle and the majority of their properties are very sensitive to long exposure at high temperatures [14,15,16,17]. This is because the polymers acting as binders in the PC are organic substances, which exhibits lower resistant to heat than the inorganic ones. Accordingly, PC is not recommended to be used in prolonged high thermal exposure to due to it generates the degradation of the resin, which possibly results in a loss of mechanical and fracture strength. These drawbacks are limiting the extensive use of PC as becoming not more efficient option [18,19].

Several studies have been undertaken to improve the PC performance throughout the insertion of flame retardant, coupling agent, and fibers, or by the replacement of the solid part by industrial by-products (i.e., industrial wastes, recycle aggregates, etc.) [2,4,20,21,22,23]. However, few studies have been performed to investigate the retaining of mechanical and fracture properties of PC after long exposure in extreme thermal conditions. Elalaoui et al. showed that the mechanical and physical PC containing epoxy was obviously influenced by thermal conditions but still more efficient than Portland cement concrete even after exposure to temperatures around 250 °C [13]. Apart from the mechanical and fracture properties, PC composite incorporating epoxy as main binder material are relatively more expensive than composite made with other thermosets such as polyesters [9,24]. Regardless their high cost ranging between three and fifteen times more than other thermosets, epoxy resins are preferred in special applications where high cost can be easily balanced by their overall superior mechanical and fatigue properties [7,11,25,26].

The inclusion of synthetic or natural fibers seems to be an effective option to overcome the PC shortcoming and increase the strength capacity, ductility, and toughness of PC [27,28]. G.Martínez et al. [27] investigated the effects of adding polypropylene fibers on compressive properties of polymer concrete. Their results revealed that compressive of PC reinforced with fibers was improved. Reis et al. studied the effect of reinforcing epoxy PC by chopped glass (inorganic) or carbon fibers (organic) with different proportions [11,29]. A similar approach was taken by Rokbi et al. [30] who tried to valorize the use of natural resources by using laminated vegetable fibers consisting of woven fabric jute in various orientations to lead up the reduction in the environmental impact and improve the mechanical properties of PC. In both studies, it was shown clearly that the fractures, elasticity modulus, compressive, and splitting tensile and flexural strength of PC reinforced with fibers were improved.

Many models have been used to predict the crack propagation for different materials [29] such as non-linear elastic fracture mechanics, linear elastic fracture mechanics (LEFM), fictitious crack model (FCM), effective crack model (ECM), cohesive crack model (CCM), size-effect model, and two-parameter model (TPM). It was reported that the carbon fiber reinforcement improves the toughness of the PC by 29%, while the glass fibers generate a smaller improvement of 13%. The tenacity of PC is 36% higher than that of Portland cement concrete which is between 0.74 and 1.53 [11,30]. The last finding indicates that the PC is more resistant to crack widening even without reinforcements.

The TPM developed by Jenq and Shah [31] and based on determining the change in potential energy when crack extents will be used in this study as it is considered as one of the fracture models to implement [11].

A review of recent literature showed a considerable number of studies on polymer PC. However, limited work has been conducted to investigate the mechanical and fracture properties of fiber reinforced polymer concrete, which undergoes extreme thermal exposure conditions. Hence, the main objective of this study is to evaluate the degradation and the retention of the residual mechanical characteristics and toughness of epoxy-based polymer concrete reinforced randomly with short carbon and polypropylene fibers after exposure to elevated temperatures. In this study, short fibers were added in a rate of 1 and 2% by the total weight of the PC composite. The exposure temperatures cycles used were ranged between 23 to 250 °C. Various tests were conducted including flexure strength, elastic modulus, toughness, tensile crack opening, density, and porosity to evaluate the effect of addition of short fibers on fracture properties of PC.

## 2. Materials, Sample Preparation and Methods

### 2.1. Materials

A thermosetting two-component product (hardener and resin) was selected to prepare the polymer concrete. Commercialized epoxy resin under the reference EPONAL 371 obtained from Bostik SA (France) was used in this study. The epoxy was cross-linked with chemical modified polyamines hardener. It was mixed immediately before use for common applications. Advantageously, the curing of the selected thermosetting resin can be implemented at a temperature at 10–35 °C, but preferably at room temperatures (20–25 °C) as prescribed. The service temperature range of this epoxy has not been mentioned in technical data but the glass transition temperature was 82.51 °C, as measured using a differential scanning calorimetry machine TA Instruments Q100 DSC (Waters LLC, New Castle, UK). The epoxy properties are presented in Table 1.

Two kinds of fibers (carbon and polypropylene) were chosen to be embedded into the PC’s mixes (Table 1). A carbon fiber marketed as Aceca^®^ ECO-H6 was supplied by Torayca Company, Japan. These fibers (having cylindrical shapes) are chopped into short lengths in the interest to help dispersion in viscous fluids. The second type of fiber, labeled under the name of DUOMIX FIRE^®^ (M6), was provided by Bekaert (Belgium) as fine monofilaments polypropylene. These fibers have a tubular shape and come in the form of clusters which can be easily dispersed during mixing. The main disadvantage of these types of fibers was lowering the workability of the mix for the same volume of polymer.

Sand graded from 0 to 4 mm and a gravel graded from 4 to 10 mm were used as fine and coarse aggregates, respectively. The specific gravity of the sand was 2.47, whereas the specific gravity of gravel was 2.53 g/cm^3^. Preliminary investigations based on the compressible packing model (CPM) was conducted to obtain the proportions of aggregates that guaranteed the maximum compactness of solid grains while minimizing the amount of polymer needed to wet fully the aggregates and fill gaps between. Portland cement CEM I 52.5 N having a specific density of 3.13 g/cm^3^ and a specific surface equal to 3590 cm^2^/g as evaluated via Blaine apparatus was also used in this study.

### 2.2. Sample Preparation

In this study, four PC mixes with fibers reinforcements and 13% of polymer, by weight of PC, were prepared. The PC samples were prepared according to a process described in the work of Elalaoui et al. [13].

The content of polymer was kept constant for the all mixes. This content was selected as the optimal amount in the basis of previous studies [13,25,32,33,34,35]. The fibers were added at the rate of 1% and 2%, by the total weight of the polymer. The PC compositions are listed in Table 2. The formed PC mixes are named as PC-Xp, where Xp% denotes the type of reinforcement (C for carbon and P for polypropylene) and p referring to the mass fraction of the introduced fibers in term of percentage. The PC was casted according to a well-defined process as described in a previous published study [13]. Ordinary Portland cement concrete (OCC) samples were also casted by mixing the same aggregates proportions for comparison purposes. The OCC mix constituent is shown in Table 2.

### 2.3. Testing Method

Several tests were conducted including compression and flexure strength, elastic modulus determined using the ultrasonic pulse velocity method (UPV), toughness, tensile crack opening, density, and porosity (using mercury intrusion porosimetry, MIP) to evaluate the effect of addition of short fibers on fracture properties of PC. The principle of MIP is to inject a no-wetting liquid such as mercury inside the pores of a small sample (10 cm^2^ as maximum) under vacuum conditions and under pressure ranging from 14 to 414 MPa.

A uniaxial compression tests were carried out using cylinders sample (50 mm × 100 mm). The effect of adding fibers on the flexure strength, toughness, and mechanical residual properties of PC were investigated by performing three-point bending test beam (50 mm × 50 mm × 305 mm). These tests were conducted in a displacement-control mode with a constant rate of 1.0 mm/min and 1.25 mm/min for bending and compression, respectively, as per the procedure stated in RILEM PCM8 1995 [36].

**Figure 1:**
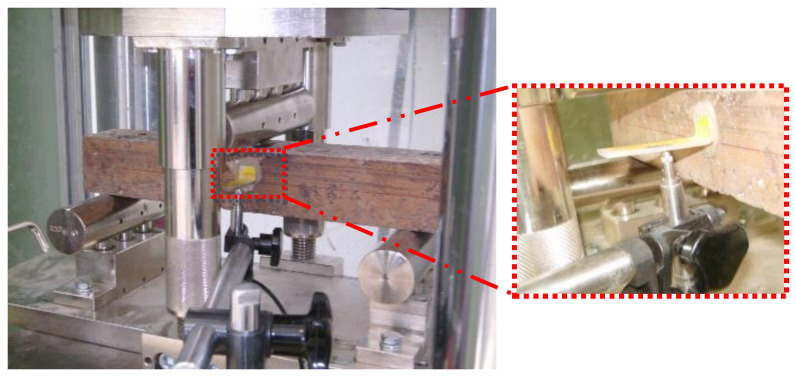
Details of bending test apparatus.

The midpoint deflection of beams was evaluated by the mean of a linear variable differential transformer (LVTD) rested on a steel L-shape bracket attached to specimens at mid-height as shown in the figure below (Figure 1).

**Figure 2:**
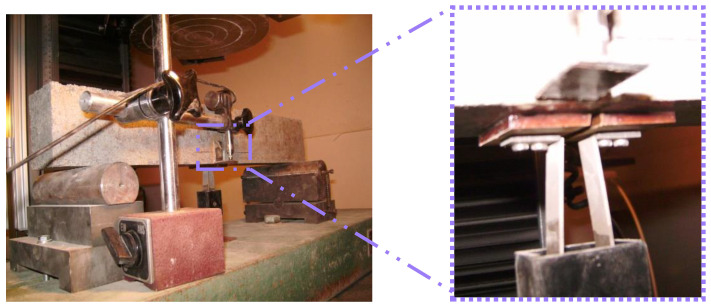
Experimental setup for CMOD controlled three-point bending test.

Concrete toughness was evaluated throughout pre-notched beams using a 250 kN closed loop INSTRON machine. The crack mouth opening displacement (CMOD) was measured by virtue of clip-gauge attached to a knife-edges installed at the bottom of the beams (Figure 2). The mid-span deflection was recorded using an LVDT placed at the left side at the bottom of the specimens. The dimensions of sample and central U-shape notch properties are itemized in Table 3.

For the OCC mix, beam samples of 70 mm × 70 mm × 280 mm for bending test and cylinder samples of 150 mm × 300 mm for compression sample were casted. The OCC beams samples were loaded at rate of 0.05 MPa/s, whereas the cylinder samples was loaded at 0.5 MPa/s.

The fracture toughness values of PC in mode-I, KIC, and the fracture energy GF were calculated using a two fracture parameters model [37,38] which appears to give rather realistic prediction of concrete fracture behavior. An actual crack is replaced by an equivalent fictitious crack [39].

The effective crack length and the peak load, are required to determine K_Ic_ using linear elastic fracture mechanics, LEFM. This value of K_Ic_ was found to be independent of the specimen size [40].

The stress intensity factor is given by means of Equation (1):

(1) $$K_{\mathrm{Ic}}{=\sigma }_{\mathrm{NC}}\;\sqrt{{\pi a}_{c}}{\;F}_{1}\left(\alpha \right)$$

where $\sigma_{\mathrm{NC}}=\frac{3F.S}{2b{.d}^{2}}$ and $\alpha =\frac{a_{c}{+d}_{0}}{{d+d}_{0}}$

(2) $$F_{1}\left(\alpha \right)=\frac{1.83-1.85\alpha +4{.76\alpha }^{2}-5{.3\alpha }^{3}+2{.51\alpha }^{4}}{\left(1+2\alpha \right){\left(1-\alpha \right)}^{\frac{3}{2}}}$$

The critical crack length is presented by Equation (3)

(3) $$a_{c}=a_{0}\frac{C_{u}{\;f}_{2}\left(\alpha_{0}\right)}{{\;C}_{i}{\;f}_{2}\left(\alpha \right)}\;\;\mathrm{and}\;\alpha_{0}\frac{a_{0}}{d}$$

**Figure 3:**
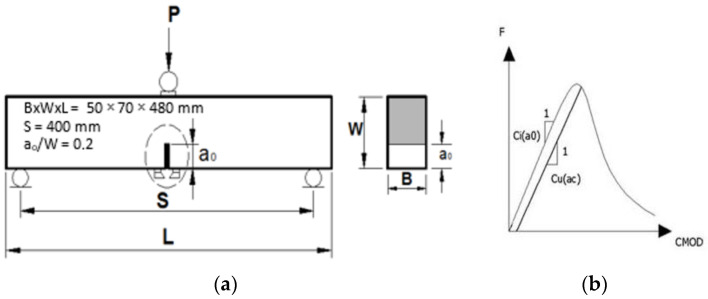
Determination of fracture parameters of concrete (**a**) geometrically characteristic values, (**b**) typical F-CMOD curve.

The geometrical parameters S, b, d, a_0_ and d_0_ are described in Figure 3.

The fracture energy is calculated according to the following relation as postulated by the following LEFM equation:

(4) $$K_{\mathrm{Ic}}=\sqrt{E{.G}_{F}}$$

**Figure 4:**
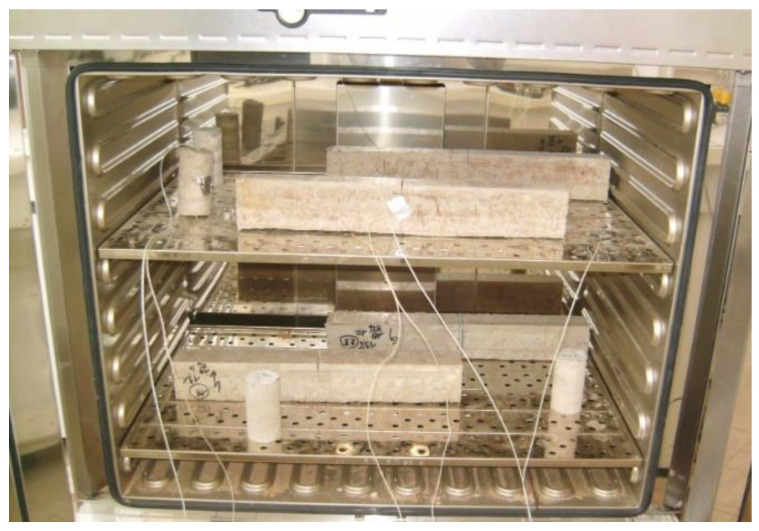
Temperatures control through installation of thermocouples.

The prepared specimens were heated at a rate of 0.5 °C/min until target temperature was reached according to the predefined protocol. The temperature was maintained for 3 h before being decreases with the same heating rate till samples reached the ambient temperature being kept for 24 h until the specimens were tested. To ensure that the setting temperature (from 100 °C to 250 °C) is compatible with specimen temperature, two k-type thermocouples mounted on data logger were attached to one side and in the middle of samples for a continuous recording (Figure 4).

A set of four specimens was allocated for each test type. The tests were conducted on concretes allowed to cure for 7 days at prescribed room temperature for both unreinforced and reinforced PC and 28 days age for OCC.

**Figure 5:**
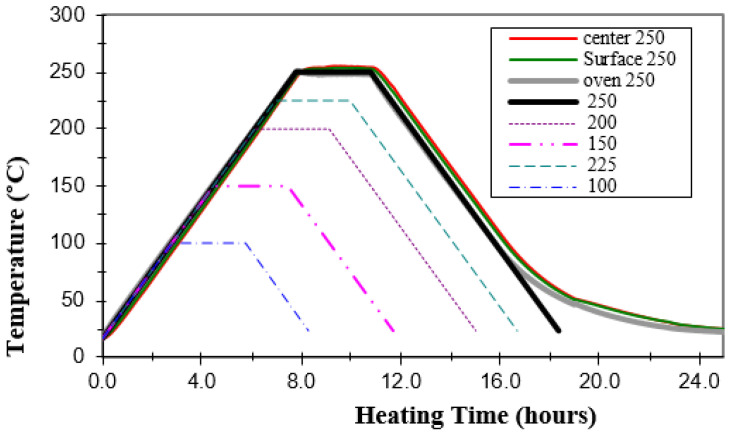
Real time-Experimental temperature records vs. heating time.

The setting and recorded temperatures were close and only a difference not exceeding ±3 °C was reported (Figure 5).

## 3. Results and Discussion

### 3.1. Fibers Adding Effects on the Density and Porosity Properties of PC

The effect of addition of short fibers on the density PC mixed is shown in Table 4. The results reveals that the inclusion of short fibers have a slight effect on the density of PC concrete. The density of the studied mixes ranged from 2.16 g/cm^3^ to 2.23 g/cm^3^. In fact, a marginal decrease was observed for the entire mixes. The same trend can be observed for the carbon group of mixes as the density decreased with higher fiber dosages. The maximum difference in densities recorded over the mixes was 3.2%, which shows that incorporation of fibers with small quantities did not significantly affect the concrete density. In addition, it was noted that the density of polypropylene fibers is lower than that of polymer. This could be due to the deficiency of uniform distribution during the mixing process and thereby in concrete bulk.

**Figure 6:**
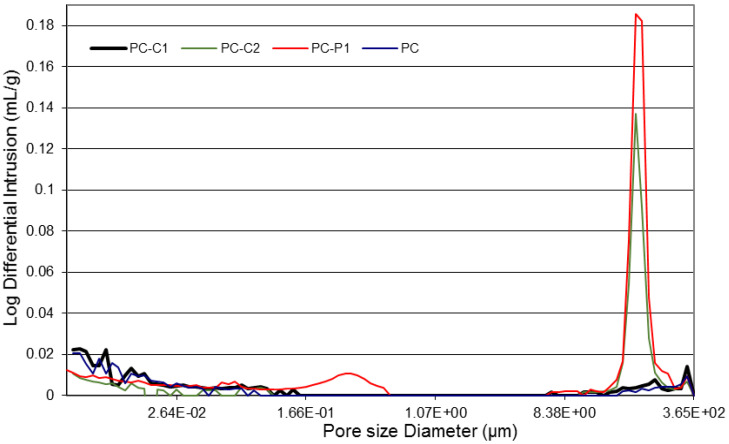
Pores size distribution for different PC mixes.

The pore structures (i.e., mean pore size, pore size distribution, and various pores size proportions) were characterized by means of MPI and results are presented in Table 4. The introduction of fibers leads also to a decrease in the mean most distributed pore diameter dc. The increase in the porosity of PC reinforced with polypropylene fibers can be attributed to volume expansion of the fibers during mixing which conducts to an additional porosity. In general, the addition of short fibers to polymer concrete generates difficulties of the solid particles compaction causing an increase in the total porosity and a decrease in the most distributed pore diameter, d_c_ as shown in Figure 6. Similar phenomena have been observed by Bentur et al. [41] and Barbuta et al. [42]. Although, the porosity of PC mixes increases with fiber content, but is still significantly lower than that of OCC samples.

### 3.2. Effects of Adding Fibers on the Modulus of Elasticity of PC Systems

The dynamic Young’s modulus $E_{d}$ and the shear modulus $\mu_{d}$ are determined using UPV. The two dynamic modulus are given by the following Equations (5) and (6):

(5) VL=(E(1−ϑ)ρ(1+ϑ)(1−2ϑ))12

(6) $$\mu_{d}=\rho \;V_{T}{}^{2}$$

The value of Poisson’s ratio is given by Equation (7):

(7) $$\vartheta =\frac{1-2{\left(V_{T}/V_{L}\right)}^{2}}{2-2{\left(V_{T}/V_{L}\right)}^{2}}$$

In the Equation (5) to Equtaion (6), E_d_ represents the dynamic elasticity modulus (MPa), V_L_ is the compressive P-wave velocity, V_T_ is shear wave velocity (km/s), ν is Poisson’s ratio, and ρ is the density (kg/m^3^).

**Figure 7:**
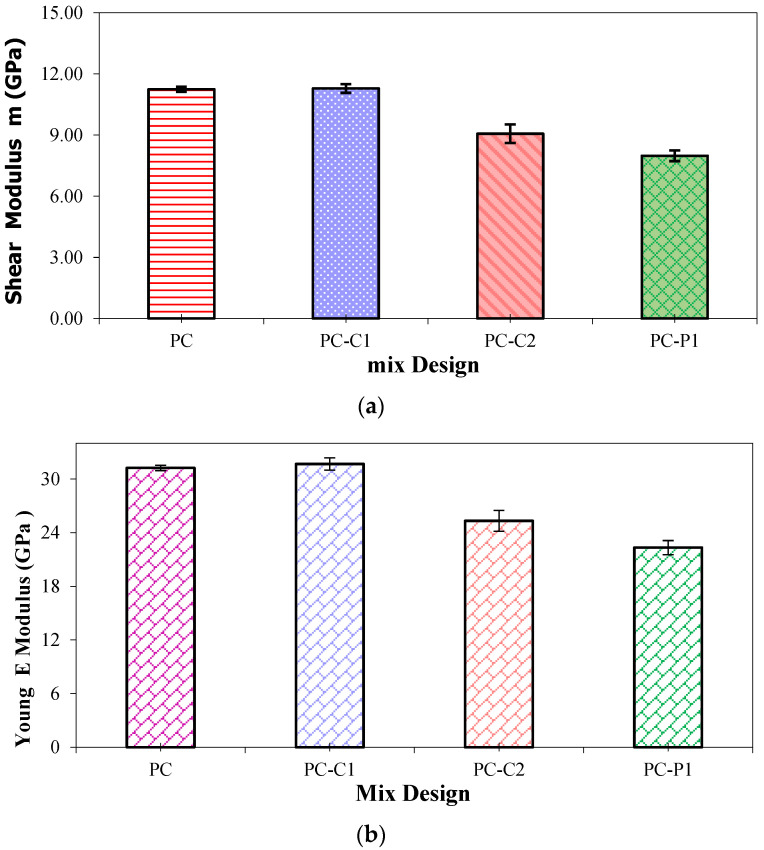
Elastic characteristics of polymer concretes: (**a**) Shear Modulus, (**b**) Young Modulus.

The results of the experimental program revealed that the static elastic modulus (Es) decreased slightly as the fiber content increased (Figure 7a,b). This marginal variation is the result of two balanced phenomena: an increase due to the high elastic modulus of the carbon fibers and decrease due to the increase in porosity and the anisotropic feature. By increasing the fraction of fibers, the growth of porosity outweighs the contribution of fibers. The lowest rigidity is observed for PC-P1 because polypropylene fibers have a low elastic modulus similar to that of the epoxy binder. Moreover, PC-P1 has higher values of the total porosity compared to control sample. In the same context, Reis and Kumar [10,43] in their research paper confirmed that adding carbon and glass fibers do not improve the compressive elastic modulus of the composites but in opposite slight decrease was observed while carbon fiber reinforcements are incorporated.

**Figure 8:**
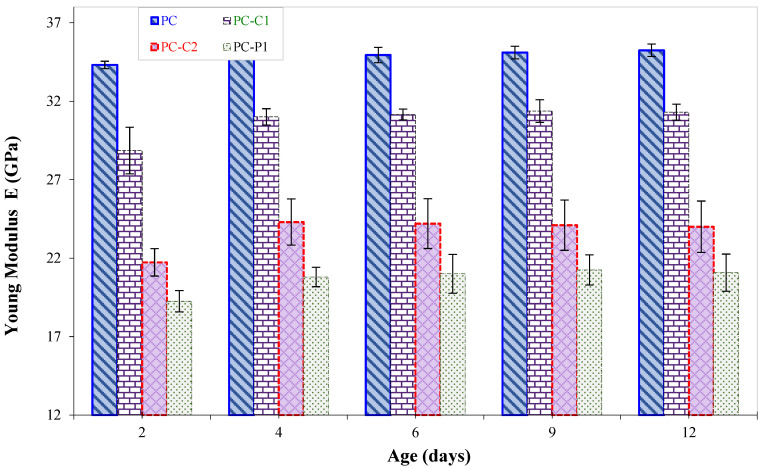
Elastic modulus vs. age for PC mixes.

By adding fibers to PC, all mixes showed indistinctly an increase in the modulus with sample age and being stabilized after a period of 5 to 7 days (Figure 8). This can be attributed to the progress of the curing reaction taken end at the 7 days as maximum leading to the formation of polymeric epoxy structures [16]. It should be mentioned that the modulus of elasticity records for unreinforced PC displayed are in accordance with the values described in literature for epoxy-based PC [11,44].

### 3.3. Fibers Adding Effects on the Mechanical Properties of PC

**Figure 9:**
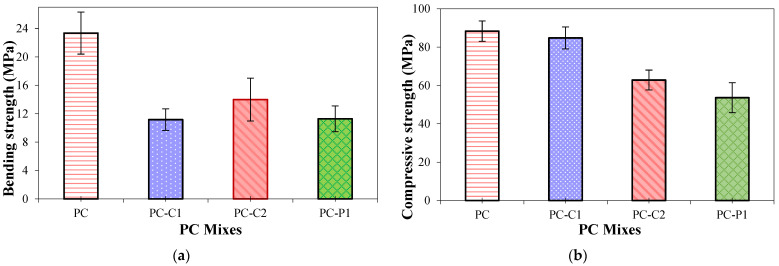
Bending and compressive resistances of different PC sets: (**a**) Flexural strength, (**b**) Compressive strength.

The results of flexural and compressive strength of PC mixes are presented in Figure 9a,b. The composite systems exhibit lower flexural and compressive characteristics when reinforced with polypropylene and carbon fibers, regardless of the type of reinforcements. In fact, it was reported that polypropylene fibers as example show weak binder–fiber contacts and this decrease the performance of the final product, regardless of the binder type [45].

However, the results show that flexural strength of PC made with carbon fibers increased with the increase in fibers content (Figure 9a). In average, flexural strengths increased 20% approximately for 2% fiber content, and compressive strength decreased 25.9% for the same fiber content, comparing to PC-C1.

The decrease in the mechanical resistances is probably due to poor adhesion between the fibers and the matrix and to the high values of porosity. This decrease is a sign of decrease in transverse bonds in polymer, which decrease the stiffness and increase ductility of polymer concrete [46]. In fact, adding 1% of carbon fibers does not affect the compressive strength and the elastic characteristics but decreases the flexure strength. In other words, fibers do not increase PC strength but their addition to the mixture diminished the signs of brittleness behavior of unreinforced polymer concrete [20], which is translated by a change in bending behavior from quasi-linear brittle to nonlinear ductile, a nonlinearity heightened by increasing the fiber content.

It can be concluded that the addition of fibers did not accomplish the expected reinforce or at least has the same strength characteristics as unreinforced PC [17]. Hence, only the toughness of polymer concretes reinforced with carbon fibers will be considered thereafter.

### 3.4. Fibers Adding Effects on Toughness at High Temperatures

**Figure 10:**
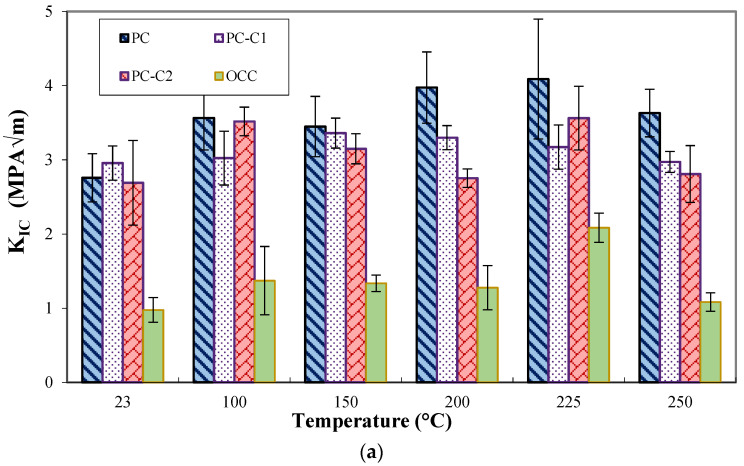

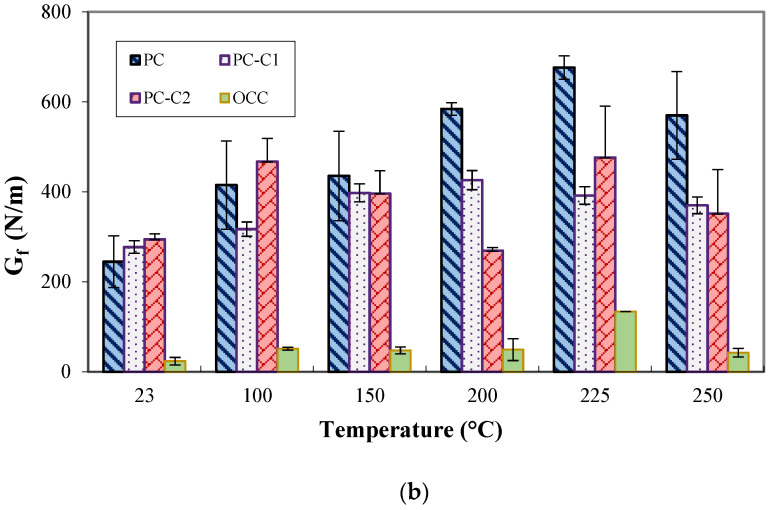
Fracture parameters as a function of heating temperature for concrete mixes. (**a**) Stress intensity factor (**b**) Fracture energy.

Figure 10a,b represent, respectively, the variation of stress intensity factor K_IC_ and the fracture energy G_F_ as a function of the exposure temperature for both the PC system and the OCC. It can be seen that PC is more resistant to crack propagation than OCC. By increasing the exposure temperature, the toughness is improved up to 225 °C for both concrete before declining slightly at higher temperatures (250 °C). Such a variation has been similarly observed for the flexural strengths mainly in the case of plain PC as a result of action of two coexistent and competitive phenomena: (i) post crosslinking initiated by heat and (ii) thermo-oxidative degradation of the epoxy polymer [47]. This variation is emphasized by a loss of bond between aggregates and the binder as demonstrated in a previous work [14].

The effect of carbon fiber introduction on the fracture properties of PC is studied and the results are depicted on Figure 10a,b. It can be seen that adding 1% of carbon fibers to polymer concretes results in an enhancement on its fracture properties at room temperature, but this effect is vanished by increasing the exposure temperature as a result of the decrease in bond strength in the fiber–matrix interface while rising temperature [14] added to the other types of degradation mentioned earlier.

**Figure 11:**
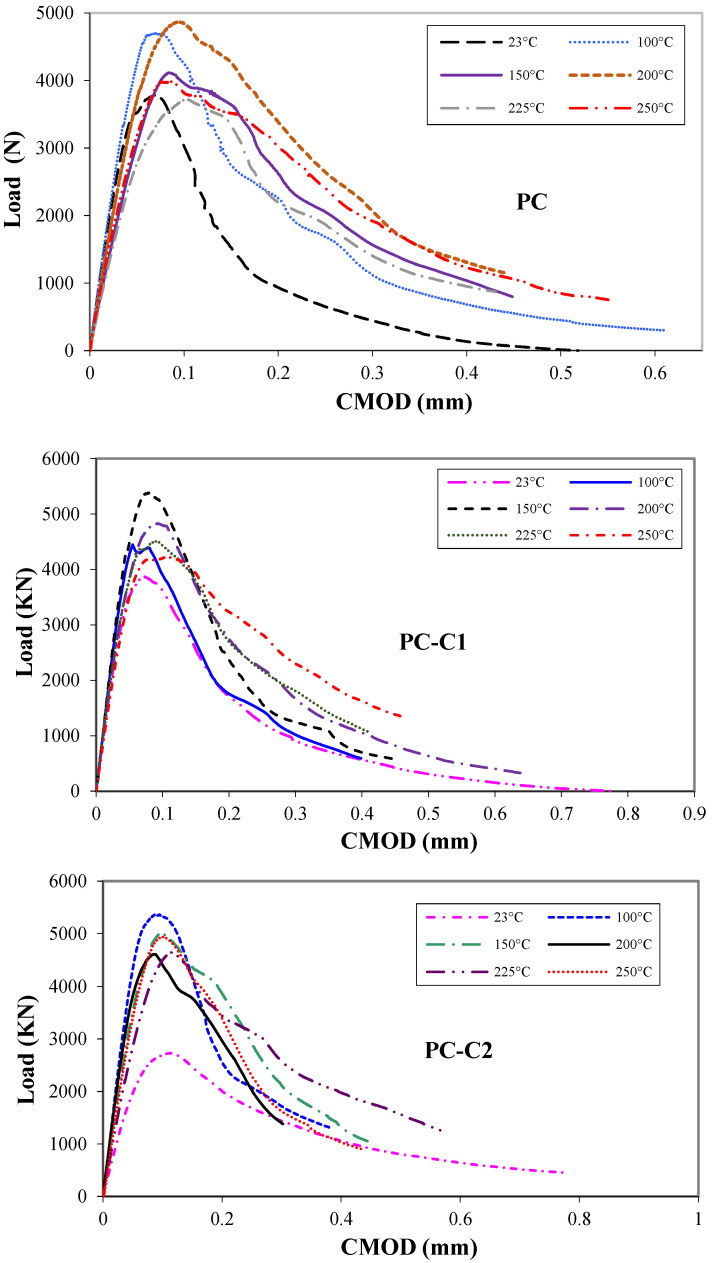
Load–CMOD for PC systems for different heating temperatures.

Figure 11 shows the load–CMOD graph for the tested notched beams as a function of heating temperature. The overall load–CMOD curves have presented a smooth softening curves after the peak loads which indicates that tests were conducted under a stable test regime. All curves observed show a similar general trend for all specimens.

It can be seen also that for reinforced PC mixes the load carrying capacity increases until 150 °C and then decreases for higher temperatures. Moreover, a significant change in the post-peak behavior was observed, the ductile behavior becomes less brittle [48]; similar to the type of behavior observed in non-reinforced concrete beams. This ductility enhancement yields slower crack propagation, a phenomenon attributed to fracture energy increase due to the existence of a micro-cracks network. It should be highlighted that more energy dissipation is necessary for the coalescence of micro-cracks into a single macro-crack [49]. The use of fine and short fibers in composite helps to reduce their critical crack length and increase fiber–matrix interface area to intercept cracks. This means that if crack passes through fibers (being less stiff than fiber), it will encounter the resin which will control and slow crack. If this does not stop crack propagation, the next fiber can intercept crack and stop it. Fibered PC force cracks to follow a very devious path that needs larger amounts of energy to create new fracture surfaces.

## 4. Conclusions

The effect of incorporating short carbon and polypropylene fibers combined with the exposure to high heating temperatures reaching 250 °C on the mechanical and fracture properties of epoxy polymer-based concrete is investigated in depth in this study and the following remarks were derived:

- Epoxy-based concrete possesses higher mechanical properties compared to ordinary cement concrete.
- For temperatures less than 250 °C, the epoxy polymer concrete is still more efficient than ordinary cement concrete.
- The addition of short carbon fibers content by a rate of 1% by weight to polymer concrete indicated that there was no indicative difference in the concrete density, the elastic characteristics, the compressive strength, and led to a decrease in the flexural strength. It also did not enhance the fracture properties at room temperature.
- When exposed to high temperatures of up to 150 °C, the 1% fiber introduction resulted in a load-carrying capacity increase. This enhancement of fracture properties is vanished for higher heating temperatures.
- Compared to the post cracking behavior at room temperature, the ductility of 1%-fibered polymer concrete increases when it is exposed to high temperatures, resulting in slower crack propagation.
- Carbon fibers introduction at content of 2% by weight of polymer-based concrete did not improve its mechanical and fracture properties. The same was observed for polypropylene fibers used with a fraction of 1% by weight.

## 5. Recommendations

Due to complexity and co-existence of many phenomena, deeper investigations are hence needed to explain their interaction that affect the properties of reinforced fiber polymer concrete. Curing conditions of samples and their effects in the fracture and thermo-mechanical properties seems to be interesting to be investigated and evaluated to build a complete and consistent knowledge about polymer concrete exposed to extreme in-service conditions.

## Figures and Tables

**Table 1:** Properties of used materials according to the manufacturer.

Properties	EPONAL 371	Polypropylene Duomix^®^ Fire M6	Aceca^®^ ECO-H6
Tensile strength (MPa)	31.7 ± 3.2	300	3530
Young’s modulus (MPa)	3800 ± 130	3500–3900	230
Elongation at break (%)	1.2 ± 03	15	1.7
Compressive strength (MPa)	81.05± 8.9	–	–
Length (mm)	–	6	3
Density at 23 °C (g/cm^3^)	1.42–1.48	0.91	1.73–1.96
Color	Beige	transparent white	black
Brookfield viscosity at 23 °C (Pa.S)	5-12	–	–
Filament diameter (µm)	–	18	7
Melting temperature (°C)	–	160-165	3500

**Table 2:** PC and OCC mixes with the varying fiber’s content.

Material	Concrete Mix				
	PC	PC-C1	PC-C2	PC-P1	OCC
Polymer (%)	13	13	13	13	-
Cement CEM I 52.5 (%)	–	–	–	-	15.6
Sand (%)	56.6	55.6	54.6	55.6	35.1
Gravel (%)	30.4	30.4	30.4	30.4	41
Water	–	–	–	–	8.3
Carbon fibers (%)	–	1	2	–	–
Polypropylene fibers (%)	–	–	–	1	–

**Table 3:** Dimensions of sample and central notch properties.

	Beam Dimensions b × d × L	Rete of Loading (mm/min)	Span Length S	Length of the Notch ao	Width of the Notch	$\frac{a_{0}}{d}$
PC	50 × 70 × 480	0.05	400	14	3 max	0.200
OCC	80 × 150 × 750	0.05	640	48	3 max	0.312

**Table 4:** Density and porous structure properties of studied concretes.

Concrete System	Density (g/cm^3^)	Total Porosity (%)	d_c_ (µm)
PC	2.23	3.6	175.4
PC-C1	2.20	4.6	83.8
PC-C2	2.17	8.8	51.9
PC-P1	2.16	12.30	61.0
OCC	2.37	15.13	–

## Data Availability

Not applicable.
